# Molecular Detection of *Bartonella alsatica* in Rabbit Fleas, France

**DOI:** 10.3201/eid1612.100696

**Published:** 2010-12

**Authors:** Tahar Kernif, Philippe Parola, Jean-Claude Ricci, Didier Raoult, Jean-Marc Rolain

**Affiliations:** Author affiliations: Université de la Méditerranée, Marseille, France (T. Kernif, P. Parola, D. Raoult, J.-M. Rolain);; Institut Méditerranéen du Patrimoine Cynégétique et Faunistique, Vergèze, France (J.-C. Ricci)

**Keywords:** Vector-borne infections, bacteria, zoonoses, Bartonella alsatica, fleas, rabbits, France, letter

**To the Editor:** *Bartonella alsatica* was first isolated from the blood of wild rabbits from the Alsace region in France (*1*). This bacterium is now considered an emerging infectious disease zoonotic agent in persons in close contact with rabbits; at least 2 human cases of endocarditis and 1 human case of lymphadenitis have been reported (*2**–**4*). In this study, we report the molecular detection of *B*. *alsatica* in fleas (*Spilopsyllus cuniculi*) collected from rabbits in southern France.

During January and February 2008, a total of 60 fleas were collected from wild rabbits (*Oryctolagus cuniculus)* from 3 regions in southern France: Canohes (42°38′N, 2°51′E), Pollestres (42°38′N, 2°52′E), and Toreilles (42°45′N, 2°58′E). The fleas were collected and identified phenotypically, kept in ethanol, and sent to Unité de Recherche sur les Maladies Infectieuses et Tropicales Emergentes in September 2009.

DNA from these fleas, as well as negative controls from uninfected lice maintained as colonies in our laboratory, were extracted by using a QIAmp Tissue Kit (QIAGEN, Hilden, Germany), as described (*5*). Identification of flea species at the molecular level was achieved by PCR amplification and sequencing of partial siphonapteran 18S rDNA gene (1.95 kbp) as described (*5*). Sequences were assembled in Sequencher 4.2 (GeneCodes Corporation, Ann Arbor, MI, USA). DNA was used as templates in a real-time quantitative PCR specific for a portion of the *Bartonella* genus 16S–23S intergenic spacer (ITS) performed in a Smart cycler instrument (Cepheid, Sunnyvale, CA, USA), as described (*2*). Positive samples at the genus level were confirmed by PCR amplification and sequencing of the *Bartonella* ITS region, as described (*2*). Finally, *B. alsatica* amplification and specific identification was confirmed by using 2 new specific PCRs with primers and TaqMan probes (Applied Biosystems, Courtaboeuf, France) specific for a portion of the heat shock protein 60 (*hsp60*) and the DNA gyrase subunit B (*gyrB*) genes of *B. alsatica* (Table). Specificity of these 2 PCRs was verified in silico (computer simulation) and by using a panel of 14 *Bartonella* species available in our laboratory (data not shown).

**Table:** Oligonucleotide primers and TaqMan* fluorescent probe sequences of *hsp*60 and *gyr*B genes used for reverse transcription PCRs of *Bartonella alsatica*†

Gene	Oligonucleotide	Sequence (5′ → 3′)	Length, bp	Amplicon size, bp
*hsp60*	B_alsa_hsp60_F	TGCTAACGCTATGGAAAAAGTTG	23	108
	B_alsa_hsp60_R	CCACGATCAAACTGCATTCC	20	
	B_alsa_hsp60_P	6FAM-TGTCGAAGAAGCAAAAACGGCTGAAACC-TAMRA	28	
*gyrB*	B_alsa_gyrB_F	CGAAGCAAAACTTCTTATTAGTAAGGT	27	126
	B_alsa_gyrB_R	GCAAGCTTTCCTGGCAGAG	19	
	B_alsa_gyrB_P	6FAM-ATAGAGGCTGCTGCGGCGCG-TAMRA	20	

*Applied Biosystems, Courtaboeuf, France. †*hsp60*, heat shock protein 60; gyrB, DNA gyrase subunit B.

All fleas were morphologically identified as *S. cuniculi* by using current taxonomic criteria (*6*). Moreover, the 18S rRNA gene amplified and sequenced as described (*6*) from fleas gave a sequence with 100% similarity with the sequence of *S. cuniculi* fleas deposited in GenBank (accession no. EU336097). *B. alsatica* was detected by ITS reverse transcription–PCR in 8 (13.3%) of 60 fleas: 6 from Toreilles (17.6%, 6/34) region, 2 from Canohes (10.5%; 2/19), and none from Pollestres (0/7). Sequences obtained after PCR amplification and sequencing of partial ITS showed 96.6% identity with *B. alsatica* (GenBank accession no. HM060955). Using our 2 new PCRs specific for partial *hsp60* and *gyrB* genes from *B. alsatica,* we identified all *Bartonella* spp.–positive fleas, which had cycle threshold values ranging from 12.15 to <32.35 and 13.21 to <36.99 for *hsp60* and *gyr*B genes, respectively.

We report the specific detection of *B. alsatica* in *S. cuniculi* rabbit fleas from southern France using 4 different PCRs and sequencing, including 2 new reverse transcription PCRs described in this study. There is 1 report of molecular detection of *B. alsatica* from *S. cuniculi* fleas from a European wildcat (*Felis silvestris silvestris)* in Andalusia, Spain (*7*). Although *S. cuniculi* fleas are rare on cats, this study demonstrates that cats in contact with rabbits may be infected by these fleas and consequently become a potential source for *B. alsatica* transmission to humans. Márquez has also recently reported the molecular detection of *B. alsatica* in blood from 48/279 (17.2%) of wild rabbits (*O. cuniculus*) in Andalusia, Spain (*8*).

In conclusion, further research is needed to better understand the mode of transmission of *B. alsatica* in humans and mammals and the role of rabbit fleas for potential transmission for these bacteria. The recent description of *B. alsatica* as a human pathogen and the discovery of rabbit fleas as a potential vector reemphasize the emergence potential of this bacterium in humans who have close contact with rabbits.
